# A novel technique for colonic diverticular bleeding hemostasis using hemostatic forceps and the reopenable-clip over-the-line method

**DOI:** 10.1055/a-2505-9253

**Published:** 2025-01-16

**Authors:** Junki Toyoda, Tatsuma Nomura, Takanobu Mitani, Yuto Ikadai, Tomohiro Sase, Tomonori Saito, Katsumi Mukai

**Affiliations:** 1 Department of Gastroenterology, Suzuka General Hospital, Suzuka, Japan; 2 Department of Endoscopy Center, Suzuka General Hospital, Suzuka, Japan

Colonic diverticular bleeding is the most common cause of acute lower gastrointestinal bleeding. Although colorectal endoscopic submucosal dissection has spread throughout the world, and hemostasis with hemostatic forceps for the vessels within the diverticulum has become common, the use of hemostatic forceps for diverticular bleeding is not common due to the risk of perforation 1 . We propose the use of a calibrated, small-caliber tip, transparent hood with a tapered 4-mm tip (CAST hood; TOP, Tokyo, Japan) to identify bleeding in small diverticula 2 . We report a novel method of closure for small diverticular bleeding in the ascending colon using the reopenable-clip over-the-line method (ROLM), which allows complete closure of the diverticulum after postoperative vascular coagulation with hemostatic forceps ( Fig. 1 , Video 1 ) 3 .

**Fig. 1:**
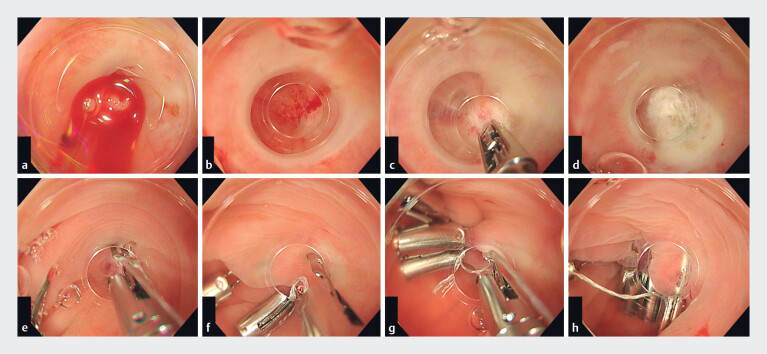
Hemostatic forceps and the reopenable-clip over-the-line method (ROLM) to achieve hemostasis in diverticular bleeding. **a** Diverticulum with active bleeding after removal of clots. **b** A bleeding point in the center of the diverticulum. **c, d** Coagulation of the bleeding point with hemostatic forceps. **e** First clip with line placed at the edge of the diverticulum. **f** A reopenable clip with a line through the tooth is placed at the contralateral defect edge. **g** A reopenable clip with a line through the tooth was repeatedly placed on the contralateral diverticular edge. **h** Diverticulum completely closed with a total of eight clips after coagulation and hemostasis of the bleeding point.

Hemostatic forceps and a reopenable-clip over-the-line method for hemostasis of diverticular bleeding.Video 1

ROLM is a defect closure method that uses a reopenable clip (SureClip 8 mm; Micro-Tech [Nanjing] Co. Ltd., Nanjing, China) and a line (0.22 mm, polyethylene line), which can be combined with a narrow 4-mm CAST hood to achieve complete mucosal defect closure even in narrow spaces 4 5 .

A 56-year-old man presented to our hospital as an outpatient with bloody stools. After preparation, a colonoscopy was performed using a CAST hood to identify the bleeding point, although no active bleeding was observed. A small diverticulum with blood clots was found in the ascending colon and active bleeding was observed after the clots were removed. Initially, a marking clip was placed near the diverticulum. Because the diverticulum was small (approximately 6 mm) and had a bleeding point at its center, hemostatic forceps were used to coagulate the blood vessels. After confirming disappearance of the bleeding point, the diverticulum was completely closed using ROLM to prevent perforation and rebleeding during the postoperative period. A clip with a line was placed at the diverticulum edge. The patient was discharged without adverse events or bleeding from the diverticulum.

Endoscopy_UCTN_Code_TTT_1AQ_2AZ

## Notes

Endoscopy E-Videos https://eref.thieme.de/e-videos
*E-Videos* is an open access online section of the journal *Endoscopy* , reporting on interesting cases and new techniques in gastroenterological endoscopy. All papers include a high-quality video and are published with a Creative Commons CC-BY license. Endoscopy E-Videos qualify for HINARI discounts and waivers and eligibility is automatically checked during the submission process. We grant 100% waivers to articles whose corresponding authors are based in Group A countries and 50% waivers to those who are based in Group B countries as classified by Research4Life (see: https://www.research4life.org/access/eligibility/ ). This section has its own submission website at https://mc.manuscriptcentral.com/e-videos .
